# Bibliographical Notices

**Published:** 1839-01-01

**Authors:** 


### Periscope;

OR,

### CIRCUMSPECTIVE REVIEW.

"Ore trahit quodcunque potest, atque addit acervo."

### Notices of some New Works.

On the successful Treatment of Consumptive Disorders, and Female Complaints connected therewith; on Scrofulous Diseases, &c. &c. By J. J. Furnival, M.D. Octavo. London, Nov. 1838.

We confess that the first two words in the title-page of this little volume excited some suspicions that another diappointment was about to be added to the many that we have experienced in a long career of practice, both at the bed-side of sickness and in the *dissecting-room* of the library. It was not unreasonable that some of the charlatanneries, couched under the seductive titles of "Consumption Curable," &c. should rise on our mental horizons, and make us sigh for the respectability of physic! From the mummeries of mesmerism, which, for six or eight months past, have excited the risibility and ridicule of every person capable of knowing his right hand from his left, but which are now laid in the Tomb of the Capulets, we must fall back upon the dull and disgusting scenes of stale, money-making, pickpocketing, homicidal quackery, unredeemed by a single touch of that sublime absurdity which rendered the animal magnetists the laughing-stocks of the profession, and the merry-Andrews of mankind! Such were the transitory reflections that passed through the mind, while cutting open the first few leaves of the little volume before us. But we very soon perceived that the very suspicion of quackery excited by the title-page, was baseless, and that the author was of a stamp that secured him effectually against enrolment in that degraded list. Dr. Furnival is evidently a man of experience, observation, learning, and general science. Indeed, if we were inclined to "hint dislike, or hesitate a fault," it would be on the score of shewing off too much erudition and knowledge in a popular work like the present, and on a subject where classical lore and general science can avail us but little, and where close clinical observation and judicious experiment are the chief, if not the only means by which we can hope to benefit the community.

Our author has evidently read a great deal on consumptive diseases, and is in possession of all that ancient observation and modern dissection could do in illuminating their pathology, etiology, and treatment. The first seven chapters embrace pathogeny, and the non-naturals, as diet, exercise, secretions, sleep, study, &c. in which the author is rather discursive—perhaps a little theoretical, or even fanciful—but that is very pardonable. The selections which the author has drawn from various sources are generally the best that could be collected, and his own observations, forming the connecting link, are judicious. There can be no doubt that, if proper hygienic precepts were carefully acted on, the ravages of consumptive diseases would be greatly curtailed.

"If due attention have been paid to the foregoing hygienic precepts—if a wholesome climate, with its purer air, have been secured—if the secretions and excretions have been attended to—if the mind have been preserved in the happy medium between inactivity and over exertion—if daily muscular exercise have not been neglected—and if, above all, the skin have been maintained in a healthy state by proper clothing, by frictions, by cleanliness, and bathings or spongings with cold water or with iodine solution—we may venture to assert that in persons so treated, the tendency towards deposition of tubercles will be counteracted, or if already deposited, there is much probability that they will be prevented from assuming an active condition; or still further, that they may be even absorbed. By these means the delicate constitution may be strengthened throughout the very important periods of childhood, adolescence, and puberty; and disease, if it do occur, may be so successfully resisted as to prevent a fatal issue."

Unfortunately the medical practitioner is rarely consulted till the phthisical disposition is formed, or even considerably advanced. And if he is resorted to for advice at an early period, that advice is not followed. Youth will not be held in trammels—fashion must have its victims—delicate young ladies must go to balls and routs half naked—to theatres when the thermometer is below 32°—and the portions of their bodies that are deemed worthy of vesture, are girded into the narrowest span that whalebone and cordage can effect.

The eighth chapter treats of the symptomatology of phthisis, and is well written, evincing accurate observation. It will be very useful to parents whose progeny are disposed to tubercular affections. The medical reader is but too well acquainted with the phenomena of consumption. The ninth is a short, but well-executed chapter on diagnosis, from which we are tempted to make an extract, as a specimen of Dr. F.'s style and manner.

"In the early periods we must first ascertain that the dyspnœa, livid face, dry cough, or else the frothy sputum streaked or dotted with blood, do not depend upon cardiac disease solely; and then we must take care not to mistake for this disease that affection of the bronchial mucous membrane called dry catarrh. In both diseases there are wasting of body, a dry cough, dyspnœa, and oppression; and these two last symptoms are exacerbated on exertion, such as running, ascending any elevation, and sometimes after meals. In both, too, these signs may remain in a latent or unchanging state for a long time, even for several years.

The oppression about the sternum and præcordia in these cases of catarrh, is also an unfailing attendant in consumption; and I doubt not but that it is connected with, if not caused by, the too carbonaceous state of the blood in this disorder.

Before the days when the light of the stethoscope was thrown upon our paths of practice, there is little doubt that these catarrhs were mistaken for tubercular consumption; but now, we can discover that percussion of the chest returns a healthy sound; and that although the respiratory murmur is, in severe cases, nearly inaudible at certain points for a time, yet it is still in most cases quite distinct; and that a slight clicking may occasionally be detected upon coughing or upon a deep inspiration. After a time the cough increases; but with judicious treatment the disease is gradually resolved, or it would have been followed by emphysema of the lungs. The various coughs, called nervous coughs (arising from hysteria, disease of the brain, &c.), stomach or liver coughs—those caused by elongated uvula, pharyngeal, laryngeal, or tracheal irritation—will, for the most part, readily be diagnosticated, by the general signs as well as by the negative results, in respect of the lungs, afforded by the stethoscope.

Chronic or senile bronchitis may be mistaken for phthisis in its latter stages, when vomicæ have formed; the opake, yellowish or dirty green, pelletty sputa of bronchitis being not altogether unlike the tuberculous matter; but in complicated bronchitis, the history of the disease will inform us that the lower lobes were first or simultaneously affected; and also, that there has been a mucous wheeze, together with considerable distinctness of the respiratory murmur from the beginning."

The tenth and last chapter is on the treatment of phthisical diseases, accompanied by a few select cases. The principles of treatment—at least of preventive treatment—may be easily gathered from the following few lines.

"From the preceding observations, it may be collected, what means must be adopted with a view to strengthen the constitution of a person who is in delicate health; and that those means are directed towards inducing a healthy state of every organ, by promoting the due performance of every function in the human body. The prominent tendencies in the weak or invalid constitution, seem to consist in, or to produce, an imperfect action or debility of the capillary system—a torpor of the cutaneous functions—internal congestions (venous)—and an arterial blood not sufficiently decarbonized. These states or conditions of the human animal economy, if suffered to remain unchecked would soon lead on to disease, most serious as to character, most threatening to life, and very intractable as to treatment."

This principle of prophylaxis applies to almost every disease in the nosology. It applies to the cure, too, as well as the prevention. If we can bring every organ and function into a healthy state, we shall have no disease to debar or cure. The worst of it is that such a state is never seen—even in the most apparent health—and can never be induced in such a class of diseases as that of consumptive—or indeed of any class. In the scrofulous and phthisical constitution we can seldom keep even the majority of functions in a state of health. All we can do, then, and all that we can expect, is to lessen as much as possible the derangements of function in the several organs, since we well know how little power we have over organic changes. This minute attention to all the functions, and exact regulation of all the non-naturals, is the "successful" method of treating consumptive diseases announced by our author—and doubtless it is the most rational and the most effective plan of treatment. Dr. F. proclaims no specific—no *inspired* nostrums—no lung-stretchers—no infallible liniments or other quackeries. The following passage may be easily applied where the shoe pinches.

"One cannot but be aware of the danger which now exists, of overrating the influence of remedies in phthisis. Since the time when Laennec discovered the cicatrization, or fibro-cartilaginous lining of cavities left by vomicæ: and when he, in prophetic, as well as strictly philosophical spirit, boldly announced the future *curability of consumption*, many persons (*some, perchance, with motives not altogether above suspicion*) have raised a cuckoo-note, and proclaimed themselves consumption-curers, even in advanced stages of the disease;—with stethoscope in hand, they profess that they can watch, and accurately tell, to what extent each cavity fills up, *from day to day*. With such persons, the faithful inquirer cannot coincide. For although we may effect a great deal of good in the early stages; and although we occasionally meet with extraordinary instances of suspension of morbid action in the more advanced periods; yet in these latter, when disorganization has ensued to a considerable extent, the medical practitioner can do little or nothing as to cure, though he can help much as to palliation."

The abominable length to which the Charlatannerie abovementioned is now carried by certain unprincipled practitioners, is truly disgusting. These vampires have no hesitation in assuring their patients and the relations of their patients, even a day or two before death, that the excavations are filling up rapidly, and that recovery is certain! What care they about the disappointments—bitter ones—which the friends are doomed to experience. They know that the mouths of their patients will be sealed by the hand of death, and that the friends will be ashamed to proclaim the quackeries of the doctor, as reflecting, in some degree, on those who employed and believed him.

We must close our notice of this respectable little volume with one more extract.

"Fortunately for those whose purses will not allow of travelling and sojourning in foreign lands, but very few if any cases will now imperatively require to be sent abroad; for lately a most ingeniously constructed and practically useful apparatus has been offered to the public, the objects of which are to enable the pulmonary or consumptive invalid to take exercise, without risk, in the open air as frequently as may be necessary, and to prevent the bad effects arising in the respiratory passages from sudden changes in the temperature. I allude to the Oral Respirator.

One of the great benefits afforded by a warm climate consisted in this, that it allowed the invalid to take exercise and walk about in the open air, at less risk and with less coughing than was possible in a colder or more variable climate; but from a residence of some years in a warm climate, whither invalids were wont to flock, I am convinced this advantage was not always obtained, and that many a piercing change of weather, incidental to such climates, was unnoticed or disregarded by writers, who recommended these migrations.

However this may be, this benefit is now offered to the Englishman in the Respirator; and he may enjoy every possible home comfort, at the same time, that by doing so he does not throw away any of his chances of recovery. Such is my high opinion of this instrument, after having worn it, and after having seen how it has acted with several of my patients, that I would now with great confidence of success, enter on the treatment of many a case of pulmonary consumption, and of irritable air-passages, which hitherto I should have deemed it my duty to send to Madeira or elsewhere."

The use of the respirator is becoming daily more extended and appreciated. It will enable thousands to pursue their avocations in the open air, who were formerly confined to their houses during a considerable portion of the year, and hundreds to remain in England, who would otherwise emigrate to Italy and Madeira. We take leave of Dr. Furnivall with sentiments of respect, and wish him success and health in his rural life, as contrasted with his metropolitan avocations.

---

ON THE IMPROVEMENT OF MEDICINE. By THEOPHILUS THOMPSON, M.D.

This little brochure formed the oration at the London Medical Society in March last, and was well received by the members. The author sets out with the increase of longevity, proved by the bills of mortality, as owing, at least in part, to improvements in medical science and practice—and this boon may be fairly granted. But he comes to closer quarters, and examines the comparative mortality of particular disease, now and heretofore. At one of the puerperal hospitals in London, half a century ago, the average of deaths in parturient females was more than one in sixty—it is now little more than one in three hundred. A similar diminution is recorded in the mortality among children. Since vaccination, the mortality from small-pox in the metropolis is reduced from five thousand to three hundred per anaum. Sea-scurvy has now nearly ceased to exist in our navies—but this is more owing to good diet, comfort, and discipline, than to lemon-juice.

Our author adduces the treatment of insanity as a proof of advancing medical science. The disease is now very generally considered as the consequence of some corporeal disorder, and, of course, the treatment is more successful than formerly. The use of the stethoscope—the diminution in number of surgical operations—the improvements in the physical education (?) of children—the attention to diet—all these, he thinks, offer indubitable proofs of the march of medical intellect.

From the future he anticipates much, and thinks that the labours of Bell, Breschet, Hall, and many others, will remove much of the veil which hangs over the mysteries of the nervous system. He particularly alludes to the investigations of Dr. Todd of Brighton, respecting an "artificial digestive fluid," by means of which he has succeeded in resolving the tubercles of phthisis into their constituent globules, thus dissipating the theories of Gendrin, Baron, Carmichael, and others. The agency of mineral waters on the minute extremities of the human tubes is expected to do great things in therapeutics. The numerical or statistical method is now being cultivated with considerable ardour and expectation.

"No one can too much admire the noble, self-denying contempt of labour, or the comprehensive talent of Louis, the founder of the numerical school, leaving the lucrative engagement of an established practice, and devoting himself, in the hospitals of Paris, to a rigid investigation of medical doctrines; but what are the practical results of his exertions? To mention one of the most prominent illustrations: he has detailed a number of cases of inflammation of the lungs, giving the numbers treated by various methods and the proportions recovering, under each plan, with a view to determine the comparative efficacy of different modes of practice, especially bleeding, and the conclusion is, that in pulmonary inflammation the *power of bleeding is very limited;* in short, that it is *almost indifferent whether we bleed or not.* You will at once perceive, Sir, the fallacy of the conclusion. We know, from our own experience, that in pneumonia, a free use of the lancet (with the aid of antimony) is generally followed by a cure; and some of us, who have had occasion, on the Continent, to witness the practice of those who disapprove of bleeding, *have too often seen the disease, under such circumstances, proceed to a fatal issue.* No numerical rules can be expected to determine the treatment of diseases, which must be modified by age, sex, temperament, period, state of atmosphere, habits of life, and other circumstances, which is not in the power of figures to express. The vital principle is not amenable to numerical laws."

Yes. Those are some of the blessed effects of continental doctrines and practice. In the mode of bleeding in France, there is much less advantage gained than in that which is adopted here, and hence another reason for its deprecation amongst our neighbours there.

A great many useful hints are thrown out by Dr. Thompson respecting medical education, as tending to the improvement of medical science. He censures severely the new poor laws, as respects the medical contract system; but he has not alluded to the greatest grievance of all, and that which retards the cure of disease (which after all must be the grand object of our art) more a great deal than the new poor laws—the mode of remuneration for medical services in private life. This is the plague-spot, which medical reform writers seem afraid to contemplate or even mention! The mode of remunerating medical men for the drugs *delivered* rather than swallowed, is injurious to the patient and degrading to the practitioner. If all the medicines are taken the stomach is overloaded and sickened—if they are thrown aside—half or more of them—then the practitioner is deceived, disappointment results in that way! It is true that a few general practitioners, long established, and enjoying the full confidence of their patients, are able to charge for their visits, even under existing laws;—but the great mass of junior medici dare not risk such a step, and the evil continues. Nor will that body which regulates the education, and superintends the affairs of the general practitioners, ever try to remedy the evil in question. It is their interest to perpetuate, not to eradicate it. The more drugs that are swallowed—the more must be bought and sold—and the more grist must come to the medicine-mill in Bridge Street. Our sentiments on this point have long been known—but we confesss our astonishment at the manner in which medical reformers slur over this paramount evil, while others of very minor consequence are daily and weekly dwelt upon with all the fire of angry denunciation.

The oration is very creditable to Dr. Thompson.

### On the Causes of Epidemic Fever in the Metropolis, especially among the Labouring Classes. By Holt Yates, M.D.

This small brochure contains a meritorious attempt to draw the attention of the magistracy, and other authorities, to one of the chief causes of fever among the poor in this overgrown city; namely, malaria arising from filth of various kinds. Low fever has prevailed to such an extent, of late, that the fever-hospital cannot give admission to one-tenth of the applications, and it appears that some of the general hospitals have determined not to take in cases of fever. This is a strange resolution, and we suspect that the governors and subscribers will hardly continue to sanction it. The class of diseases in which the subscribers are most interested, whether occurring among their own servants, or their poor neighbours—is fever. Besides, as every inflammation of an internal organ is attended by *fever*, how will the nature and seat of the malady be ascertained till after reception? Will the diagnosis be left, on all except receiving days, to the discrimination of the apothecary? Or, if no topical inflammation be apparent, is the poor wretch to be turned from the hospital as a dangerous inmate labouring under typhus? The regulation is monstrous!

"The hospitals have now come to the determination not to admit, in future, fever cases at all; and I would ask what, under these circumstances, are the industrious classes to do? However ill they may be, they cannot be taken into the workhouses. Shunned by their neighbours, perhaps, and having parted by degrees with nearly all they possess, we find them, many at least, destitute of the most common necessaries of life. They are sick and dejected; several members of the same family are to be seen lying on the floor, upon a bed of straw, with scarcely any covering, no change of linen, no nurse, no friend to help them to so much as a cup of water to slake their parching thirst; and this is not always the result of intemperance and dissipation. The most formidable disease will suddenly make its appearance, and steal insidiously from room to room, and from door to door; its course and progress may be regularly traced. I have known the same fever attack, in succession, different families that have occupied the same apartments, the result partly of contagion, and partly of the continued operation of the same causes. There is no mystery about it, nor is there any thing imaginary in it, as those will see who take the trouble to investigate the fact. I repeat, *it is the existence of these causes* which we have to thank for the prevalence of fever in the metropolis."

Dr. Yates proceeds to investigate these causes, warning the medical officers of public charities, who are obliged to visit the dens of dirt and foci of fever, to be on their guard against the pestilent air that surrounds them. He deplores the want of a "Council of Hygeia," or "Board of Health," in this country, as it exists in many parts of the Continent. We have plenty of "parish officers," "relieving officers," "visiting committees," "guardians of the poor," &c. but what is every body's business is nobody's!

The chief sources of impurities of the atmosphere are—defective ventilation—the existence of noxious vapours, dust, and other extraneous bodies—combustion, and the respiration of animals—and, lastly, the presence of disease itself. The habitations of the poor too often disclose all these different sources of malaria and febrific miasmata.

In many parts of the City there are *no sewers at all;* and the stench arising from cess-pools, privies, and drains, is often intolerable. Will it be believed, that, until within these few months, there were no sewers even in Cheapside, Fore-street, Barbican, &c.? What must be the state of the lanes, alleys, and courts, in the densely populated parts of the metropolis. The annual mortality of Middlesex, which is comparatively open and drained, has been found to be one in 36, whereas it is only one in 73 in Cardiganshire. The author proceeds to describe scenes and localities infested with fever, which would make the higher classes shudder, even at the perusal. The places of interment are mischievous sources of disease too, and are disgraceful to this metropolis. But we are unable to do justice to Dr. Yates's pamphlet, which we hope will circulate widely through the public at large, especially through those districts of the metropolis where poverty and destitution—filth and fever—so abundantly prevail.

---

Observations on the Oriental Plague and on Quarantines, &c. By John Bowring, Esq. pp. 45. Edinburgh. Tait, 1838.

The substance of this brochure was read before the British Association last year, at Newcastle. The longer we live, the more we see, and the deeper we reflect, the less faith have we in the *degree* of contagion attributed to plague, and in the efficacy of quarantines, as preventives of the diffusion of the poison. This cumbrous, costly, and vexatious system of sanatory, or rather of *insanatory* laws, is one of the worst relics of the dark ages of medicine. We very much doubt whether it ever did or ever will prevent the introduction of a single particle of plague contagion into this country. Of local origin, this pestilent fever rises every year, and prevails more or less according to season and many modifying agents. It is occasionally epidemic—and perhaps, like all endemics and epidemics, it may acquire a contagious quality, and be propagated under peculiar circumstances. But that it has neither inclination nor power to travel from the Bosphorus to the Thames, we firmly believe. What language could more accurately depict the rise and progress of the late cholera than the following, applied by an eye-witness to the plague of the East?

"I discovered at every step that the contagiousness of the plague was always *assumed*, as the groundwork of all discussions,—and that the most extraordinary absurdities,—the most amusing inventions were resorted to, in order to account for its outbreak where every precaution had been taken to avoid contact with any human being,—or any supposed infected or susceptible objects. Wherever I had occasion to witness the introduction or progress of the disease—its introduction was spontaneous,—indigenous,—endemic,—its progress never traceable from patient to patient; it broke out in districts remote from one another, between which there had been no communication, and while my own observation surrounded me on one hand with thousands and tens of thousands of cases, in which the most intimate intercourse with persons ill or dead of the plague—the dwelling in their houses—the wearing their garments—the sleeping in their beds, were not followed by disease in any shape, I was called on the other to listen to stories as evidence of the contagiousness of plague, so puerile, so ridiculous, that nothing but oriental credulity could by possibility be satisfied by them."

Some of these "oriental credulities" were actually equalled in this country, during the cholera-phobia panic of 1832-3. We shall give an instance of the oriental type.

"In an instance where a very timid person, an alarmed contagionist, who was attacked and died of the plague, had shut himself up in his chamber; it was found that his son had, for his amusement, let up a kite from the roof of the house, and it was supposed that the kite-string had been touched by a bird, which bird was imagined to come from the infected quarter of the city; the plague entered the house down the string of the kite, and the son's father became the victim."

Our readers will see, in a late Number of our northern contemporary, a paper of nearly 100 pages, on cholera in Scotland, exhibiting numerous proofs of contagion, about as forcible and philosophic as the above?

So strong is the conviction, or rather the prejudice, respecting the contagion of plague in the East, that medical men are afraid of declaring their real sentiments, lest they should be denounced, not merely as unorthodox, but as dangerous persons, who do not take the proper precautions, and the regular purifications. But even amongst the contagionist physicians of the East, the efficacy of quarantine is very much doubted. Clot Bey has thrown off the belief in contagion, and is about to publish on the subject. Most of the Egyptian physicians, too, appear to be following his example. We need hardly add that the Mahomedans, almost to a man disbelieve in the contagion of plague.

Dr. Bowring lashes severely the Lazarettoes, Boards of Health, and other appendages to the quarantine system; but as one of our contemporaries has copied nearly the whole of the pamphlet into his Journal, we need not make any extracts in this place.

We are glad to learn that the Emperor of Russia and King of Prussia have sanctioned the formation of a congress to investigate the nature of plague and the advantages or disadvantages of quarantines. The congress is to be composed of distinguished physicians and politicians, chosen by the different maritime states of Europe. Such a congress should be held in some place where plague is an annual visitor, and where authentic evidence could be obtained on the spot. The parliamentary inquiry in this country led to nothing, as few of the examined had ever seen the plague, or been in localities where it is endemic. Even of the few who had been in the East, it is very doubtful whether any of them had ever actually treated a case of plague, or done more than peep through the window or key-hole of an infected ward or lazaretto.

Dr. Bowring deserves the thanks of society at large, and of the mercantile world in particular, for this pamphlet.

---

The Medical Annual, or British Medical Almanack, 1839. Edited by *William Farr*. London. Price three shillings.

The Almanack for 1839 retains its character. It is an useful compilation. Its great aim is to further medical statistics. Mr. Farr is quite an enthusiast in that way.

It contains the usual amount of information with respect to the medical corporations, societies, hospitals, and schools. Besides these matters it presents some papers of a miscellaneous character. Of these the more remarkable are:—

Remarks on Hospital Medical Staffs, by the Rev. C. Oxenden.—History of the Medical Profession, and its influence on public health in England. By the Editor.—Statistics of the Kent and Canterbury Hospital. By John M'Divitt, M.D., Physician to the Hospital.—Facts illustrative of the Pathology of the Heart. By J. Clendinning, A.M. and M.D.—Temperance in the United States of America. By J. C. Warren, M.D., Professor of Anatomy in Boston.—On Cholera—The Laws of Mortality and Recovery in that disease. By William Farr.—Abstract of a Report of the Diseases of the Army. By Captain Tulloch. —Report of the Medical Committee of the University of London—Proposed terms of Graduation.

We shall pick out a few facts, or statements for quotation.

### 1. On Hospital Medical Staffs.

The Rev. Charles Oxenden, of Bishopsbourne Rectory, has devoted his attention to the subject of hospital medical staffs. He shews that, in provincial hospitals particularly, no constant or rational proportion obtains between the numbers of the medical staff and the patients.

"By referring," he says, "to the second and fifth columns of my annexed table A, you will find, that, while in the year 1830, when my Statistical Report of Provincial Hospitals was printed, *four* medical officers had the care of ninety-nine beds at *Leicester*, of one hundred beds at *Northampton*, and of one hundred and seventy beds at *Gloucester;* the *same number* of officers were appointed at *Hereford* to attend upon fifty-two beds, and at *Bury* upon fifty *only*. Again, while *six* medical officers were deemed ample for one hundred and five beds at *Norwich*, for one hundred and seven at *Salisbury*, for one hundred and twenty-four at *Leeds*, and for about one hundred and fifty at *Shrewsbury*;—*six* were also required for the charge of eighty-five beds at *Sheffield*, of seventy-nine at *Derby*, of sixty-five at *Canterbury*, and of sixty-two at *Bath* (General Hospital.)"

We need not follow Mr. O. in his examination of the causes of disproportions of this kind, but conclude by presenting his estimate, which appears to us a fair one, of what the medical staff of a hospital should be.

"But it will be asked, what *is* the *best* proportion for a medical staff? And this, I must allow, involves some difficulty in the reply. But though difficult, I do not think it desperate. For I am well persuaded, that, though it may be impossible to lay down a perfectly mathematical scale of proportion, adapted equally to all places and circumstances; yet it *is* possible to *approximate* to such a scale, as will be calculated to insure a *fair promise* of beneficial results. This scale, however, must, to a limited extent, be made *variable*, by reason of certain *district* peculiarities, which operate to the increase or diminution of labour. In some hospitals, for instance, *surgical* cases will be found to predominate, but in most (including I. P. *and* O. P.) *medical;* while in others they will nearly *balance*. Again, in some localities hospital disease is more generally *acute*, than in others; requiring therefore more *daily* attendance, and occupying of necessity more professional *time* during treatment, and hence justifying a more extended *division* of labour. But, as a general system for adoption, I am disposed to think that hospital staffs, formed after the following scale, would best subserve the welfare of the patients, and facilitate the operations of the boards of management. Whether, or not, it would square with the interested speculation of individuals, enters not into my calculation:—

*Proposed Number of Medical Officers for an Hospital, containing*

| | | | | | |
|---|---|---|---|---|---|
| From | 1 Bed to 75—Say | 1 Physician, | 1 Surgeon, | 1 House-Surgeon. | |
| — | 76 — 125— | 2 —— | 2 —— | 1 —— | |
| — | 126 — 175— | 3 —— | 3 —— | 1 —— | |
| — | 176 — 200— | 4 —— | 4 —— | 1 do., and 1 House Apothecary. | |
| | | &c. &c. | | | |

In those neighbourhoods (in certain manufacturing districts, for example,) where *surgical* cases predominate, I should be disposed to add one surgical officer. And when out-patients (a large majority of whom will generally be found to be *medical*) are excessive, as at Bath, an additional physician would be advantageously appointed. Under any arrangements also for visiting *home* patients, further staff appointments may become necessary. These, however, are *exceptions* to, and do not militate against the principle of, my general scale."

### 2. The Barber-Surgeons, and Surgeons not Barbers.

The following memorandum of these two classes, now happily disjoined for ever, may amuse.

Among the various uneducated classes accustomed to use edged tools, who practised surgery, the barbers were distinguished at an early period. The first charter was granted, in the 1st Edward IV., to the freemen of the mystery of barbers (Misterae Barbitonsorum) of the City of London, practising surgery (*facultate sirurgicorum*) in wounds, lesions, and other infirmities; in letting blood and drawing teeth. The alleged grounds on which the charter was granted were the ignorance, and negligence of the freemen themselves, and of other foreign surgeons (*sirurgicorum forinsecorum*) not free of the city, daily flowing into London. It is evident from this charter that in 1461 surgeons existed in London; and this is confirmed by the 32 Henry VIII., c. 42, A.D. 1540, where two distinct companies of surgeons are mentioned; the one called the Barbers of London, the other the Surgeons of London; the barbers incorporated, the surgeons without any manner of corporation. The relative position of the barbers and surgeons of London is somewhat obscure; it is illustrated by the state of things in France. Several Surgeons in Paris, with Jean Pitard, surgeon to St. Louis, at their head, separated from the faculty of medicine in 1271, and formed a distinct college; the members were allowed to marry, enjoyed the same privileges as masters of physic, and wearing the same long robes, were called *chirurgeons de robe longue*; or, to distinguish them from the barbers, *chirurgiens lettrés*. They studied medicine two years, and underwent strict examinations. The Faculty of medicine discountenanced the surgeons, and in 1505, to encourage the barbers, undertook to provide them with lectures in the French tongue. The barbers, supported by the faculty, were constantly embroiled with the surgeons; and the latter at last wearied with the strife, sacrificed themselves to the supremacy of the doctors of physic; *chirurgiens jurés* were incorporated with the *chirurgiens-barbiers*, and sank at once into insignificant rivals of the umbrageous faculty. William of Salicet, Lanfranc of Milan, Gui de Cauliac, raised surgeons to a higher rank, and better educated men than the barbers became indispensable; but these elevated surgeons attended the middle classes in sickness, and aspired higher. In England the same course seems to have been taken; the barbers, evidently supported by high authorities, attempted to suppress educated surgeons; they then tempted them into a degrading connexion, by the offer of common privileges and funds, and lands, and tenements.

### 3. Temperance Societies in Amercca.

The following statements are from the pen of Dr. Warren, Professor of Anatomy in Boston.

In 1813 a society was formed at Boston, called the Massachusetts Society for the suppression of Intemperance. The individuals who combined for this object were distinguished statesmen, clergymen, and physicians. The means employed were the annual distribution of discourses showing the great evils produced by the use of alkoholic drinks. The efforts of this society were met with ridicule and abuse for some years; their opinions, however, gradually extended among the people, and in the year 1826 the American Temperance Society was formed in the same city, and immediately began a train of active operations.

About this period, the clergy, the judges, and the medical faculty united in their opposition to alkohol. The results of all these movements appeared in the year 1835, from the following facts:—About 2,000,000 of persons who had been in the habit of using alkohol had abandoned it. More than 8,000 temperance societies had been formed, embracing 1,500,000 members. Of these societies twenty-three were state-societies, comprising all the states in the union but one. Four thousand distilleries had been stopped. More than 1,200 vessels sailed without ardent spirits, and the price of insurance lessened on these vessels. About 12,000 drunkards had been reclaimed, and more than 200,000 persons had abandoned the use of all intoxicating drinks. Since the year 1835 the numbers above stated have been increasing, and other important results have shewn themselves. The bills of mortality exhibit a decrease of deaths in the places where reform has been extensive. The inmates of poor-houses, compared with the increase of population are diminishing; the amount of crimes is decidedly less, and it is a frequent occurrence to notice in the newspapers that a county jail is without a single tenant. Alienations of property from families, whose heads had become drunkards, have lessened in a very remarkable manner in almost every town; the use of wine is diminished among the rich, and instead of the strong Spanish wines, the light wines of France and Germany are getting into general use. In consequence of this the chronic affection of the stomach, commonly called dyspepsia, which was very prevalent, has almost disappeared, and gout is scarcely heard of.

The disuse of ardent spirits in the northern states is believed to have increased the physical power of this section at least one-sixth, so that if we allow for its population about 5,000,000, the force of a million of persons will have been added, while the expense of supporting the 5,000,000, instead of being increased, has diminished by the appropriation of that grain for nutrition which was employed for distillation.

The public sentiment is so strongly in favour of prosecuting the temperance reform, that it called on the legislature of Massachusetts to prohibit the sale of ardent spirits on Sundays about a year since; and this law has operated so satisfactorily, that in the present year (1838), a law has passed prohibiting the sale of ardent spirits in less quantities than fifteen gallons, thus annihilating all the grog-shops in that state at a single blow. A similar measure has been adopted by the state of Tenessee, in the west, at a distance of 1000 miles from Massachusetts, which is in the east, and other states will probably follow their example.

---

An Account of some New Instruments for Tying Polypi of the Uterus, Nose, and Ear, and Enlarged Tonsils; with Cases. By *William Beaumont*, Surgeon to the Islington Dispensary. 4to. 3 Lithographic Plates, 1838.

The Instruments recommended by Mr. Beaumont are so ingenious, that we wish to introduce them to the notice of our readers. Yet it is impossible to render them quite intelligible without the assistance of the plates accompanying the work. These, from their size, are inadmissible. We shall therefore content ourselves with merely presenting a brief description of the more important instrument, that for tying the polypus uteri, and refer our readers to the pamphlet itself for any further information.

The instrument for tying uterine polypi, consists, among other parts, of two rami parallel to each other, save that one is slightly curved towards its point, so as to correspond in some measure with the posterior parietes of the vagina, and the more readily to allow the body of a polypus to pass between the rami; which parts of the instrument are temporarily joined together at the handle, the distance between them being capable of increase or diminution according to the size of the polypus to be tied. The curved ramus is solely for the purpose of aiding in the placing of the noose around the pedicle of the polypus, and may be removed from the rest of the instrument and from the vagina when that is accomplished. The straight ramus, besides assisting in the application of the ligature, is also, with other parts, attached to it, the means by which the noose is tightened, and rendered unyielding.

This instrument is perhaps somewhat complex, but it should be borne in mind that it is to accomplish a complex purpose. It is first to carry a noose around the pedicle of a tumor in a narrow passage; it is then to constrict the pedicle so far as to strangulate the tumor; and lastly, to jam the running end of the noose in the knot, so as to prevent any elasticity of the pedicle from enlarging the noose.

In describing Figure 2, Mr. Beaumont writes :—

"This represents the manner of arranging the ligature, which consists of a common slip-knot noose. That end of the ligature which comes from and forms the knot of the noose is represented by the uninterrupted double line; it is passed through the eye *d*, and drawn (not very tight) through the hole in the axle *k*, where it is fastened, by which means the knot of the noose is held at the very point of the straight ramus. The running end of the noose, which is marked by a double dotted line, is passsed through the eye *c*, through the hole in the axle *h*, and made fast under the spring *i*. The noose, about two inches from the knot, or more or less according to the thickness of the pedicle to be encircled, is to be placed in the eye *e* of the curved ramus, and there confined; the noose is then to be caught about two inches nearer the handle of the instrument at *b*, between the curved ramus and spring, from whence it is to be carried down to *f*, and there caught again between the same spring and ramus; it is then to be carried across to the straight ramus, and caught under the spring *g*. The several parts of the ligature parallel to the rami of the instrument are to be drawn sufficiently tight to lie close, so as not to impede the passage of the polypus between the rami.

To encircle with a ligature, by means of these instruments, the pedicle of a polypus, whether of the uterus, nose, or ear, consists in the mere act of pushing the tumor between the rami of the instrument. The application of the ligature around the neck of the polypus is then accomplished, and little else remains to be done than to tighten the noose so far as to prevent the ingress of blood into the tumor. This, I believe, may always be done at one operation, however large the pedicle; and in cases where its structure is not very firm, I think the constriction might (if it were desirable) be increased to a degree sufficient to sever the pedicle in two, for the instrument has great mechanical power, a small axle and long lever. It certainly would be a mode of excision preferable to that effected by the knife or scissors."

These extracts will give a vague idea of the general character and operation of the instrument. Anything beyond that cannot be obtained without consulting Mr. Beaumont's own account. Mr. B. is evidently an ingenious surgeon.

---

### RECENT WORKS ON SURGERY.

I. A System of Practical Surgery, with numerous explanatory Plates, the Drawings after Nature. By *John Lizars*, Professor of Surgery to the Royal College of Surgeons, and lately Senior Operating Surgeon to the Royal Infirmary of Edinburgh. 8vo. pp. 220. 18 Plates.

II. The Principles of Surgery, Volume first; containing the Doctrine and Practice, relating to Inflammation and its various Consequences, Tumors, Aneurisms, Wounds, and the States connected with them.—Volume second; comprising the Surgical Anatomy of the Human Body, and its application to Injuries and Operations. By *John Burns*, M.D. F.R.S. Regius Professor of Surgery in the University of Glasgow, &c. &c. 8vo. pp. 554-536.

It is rather singular that, at the same time, two systematic works on surgery should issue from two Northern Universities—those of Edinburgh and Glasgow. The respective authors are both men of acknowledged attainments and abilities, and their opinions on many points of doctrine and of practice would necessarily command attention. It is quite inconsistent with the nature of this Journal to analyse elementary works of this description. We can only introduce them to our readers, present an idea of their respective contents, and, perhaps, expose the sentiments of their authors on some particular points.

Let us first turn to Mr. Lizars. The present volume is only the First Part of his system. The concluding Part is advertised to appear in December.

The present Part contains—Inflammation.—Arteriotomy.—Phlebotomy.—Suppuration.—Abscess.—Ulcers.—Dissecting-room Wounds.—Mortification.—Diseases of the Arteries, Aneurism.—Of the Veins, Hemorrhage.—Of the Bones, Fractures.—Of the Joints, Luxations.—Gunshot Wounds.—Amputation.

1. *The Edinburgh College of Physicians allow their Fellows to use the Lancet, &c.*—After a succinct sketch of the history of surgery, Mr. Lizars sums up thus :—

"It is evident, that surgery has only flourished in proportion as it was strictly connected with anatomical investigation, and enriched with medicine or pathological doctrines. At first it was combined with medicine, and in the days of Herophilus, and Erasistratus, Celsus, and Galen, it was also united with medicine and anatomy. After this, when anatomy could only be prosecuted by stealth, surgery continued in a truly deplorable condition, and in the 11th century, was stigmatized by the Council of Tours. In the 12th century the school of Salernum, and also that of Naples, required only one year's study of anatomy for the diploma of surgeon. In the 15th century, we find the great Paré still a barber surgeon, and controlled by the physicians. At Oxford and Edinburgh, the two first universities in Great Britain, anatomical chairs were not established until the last century; and at neither school was dissection prosecuted until this century, and that only at Edinburgh, where the study was not sanctioned by Government till within the last six years. So that it was only at the dawn of medicine, and in the present era, that surgery, blended with physic, became a respectable profession. It is much to be regretted that there exists any separation, since it only tends to depreciate each, as physicians must be consulted in accidents, the province of the surgeon; and the surgeon must be acquainted with the treatment of fever supervening to operation. All, therefore, ought to have the same elementary education, and be qualified for every department. The operating surgeon ought to dissect daily. Lately the College of Physicians of Edinburgh have wisely rescinded a law which prohibited their fellows from using the lancet or the scalpel. 'Etenim omnes artes,' says Cicero, 'quæ ad humanitatem pertinent, habent quasi vinculum commune.'"

The College of Physicians of Edinburgh have displayed common sense and sagacity. It may be questioned whether the one or the other is to be found in the College of Pall Mall East. Recent events have shown the antiquated absurdity of *its* spirit in the person of its president.

It would be idle in us repeating what we have so often urged, that the education of the surgeon and physician should be similar. Chance, inclination, or convenience may subsequently determine the line of practice, but to that each should carry miscellaneous and common knowledge. If the physicians pursue their former system, it is not difficult to perceive that the surgeons will ultimately strip them of practice. The scholastic learning which sufficed for the descendant of Esculapius, when medicine was system, its doctrines the offspring of dogmatism and authority, and its practice little else than empiricism, is comparatively valueless in the present advanced condition of the science. Its foundations in healthy and morbid anatomy are so deep and so extensive, that whoever is best acquainted with *them* will be most thoroughly conversant with *it*. In the long run the class of men who possess the really useful knowledge will obtain its fruits, fortune and fame.

2. *Lancet Punctures in Inflammation.*

These are, probably, insufficiently estimated and resorted to. Mr. Lizars gives some short but judicious hints in relation to them.

When punctures are made, attention must be paid to the position of the part affected, for unless the return of the blood be in some degree impeded, no blood will flow. Thus when the face is the seat of the inflammation, it should be held forwards and downwards; when the scrotum, it should be immersed in hot water, while the patient stands. The lancet should be held the reverse of that while opening a vein at the bend of the arm, and the point of the instrument plunged that degree of depth corresponding with the structure of the part and the proximity of blood-vessels, and always parallel with the subjacent muscles. It should be entered here and there, or at irregular distances.

3. *Chronic Hypertrophy of the Scalp.*

We observe the following notice of this affection, which, so far as we know, is not commonly described.

There is, writes Mr. Lizars, a peculiar ulceration with chronic hypertrophy attacking the scalp, which may be considered here. It is more a thickening of the integuments than ulceration, but there are occasionally extensive sinuses, and a profuse secretion of pus, with disease of the periosteum, and even the cranium. It begins by a thickening of the integuments with achores on the surface, both of which make progress, until the skin be puffed up and divided by deep sulci, into compartments of various shapes and sizes; these latter ulcerate and communicate by sinuses, thus undermining and destroying the scalp, and involving the periosteum and even the bone. In some cases, acute pains are felt not only in the head, but all over the body. Some have laboured under rheumatism, others, syphilis. When noticed early enough, the potass destroys this morbid condition of the integuments, which then heal by means of bread and water poultices. When neglected, the sinuses must be laid open, and only partially if very extensive; the potass must still be applied, and afterwards the poultices. Alteratives, such as the sarsaparilla, or the nitric acid, should be prescribed.

4. *Linear Admeasurements for finding and securing the Femoral Artery.*

We quote the following directions for operating on the femoral artery in the case of popliteal aneurysm, because we think that linear admeasurements are valuable when precise. We know that this is questioned by some surgeons, but nevertheless we are ourselves convinced of its correctness.

"The patient should be placed on a firm table, with his feet at right angles to each other, but the affected separated from the sound limb. The space between the anterior superior spinous process of the os ilium and the spine of the os pubis is to be divided into ten proportional parts, when five and a half measured from the pubes are made the base of an equilateral triangle, which is to be constructed downwards on the thigh, the apex being distad, the base proximad; and the outer or iliac side of this triangle should be extended from the apex twice its length, when the artery will be found to run beneath this line throughout. An incision should then commence at the apex of the triangle, and be continued down the thigh to the termination of the extended line, or proportionally to the depth of skin and cellular tissue, the latter of which is often infiltrated with serum; this incision is to be deepened equal in length to the first, and should pass through the fascia lata, and this cautiously, when the pulsation of the artery will be felt; the artery is then to be denuded to the smallest possible extent of its cellular sheath; the latter of which is to be held up with the dissecting forceps in the left hand, and the scalpel in the right, with its cutting edge parallel to the vessel, and pointing outwards or fibulad."

5. *Diseases of the Bursæ and Bunion.*

"When," writes Mr. L., "the bursa over the olecranon ulnæ is distended with fluid, it may be mistaken for dropsy of the elbow-joint; when the bursa under the deltoid is diseased, there is often a communication with the shoulder-joint at the bicipital fossa; and a similar connexion as frequently exists between the bursa under the psoas tendon and the hip-joint, which renders disease of these two last bursæ very serious. The bursa superficial to the larynx is sometimes affected, and when it is allowed to rupture, sinuses form; that over the angle of the scapula now and then becomes diseased, and so also that under the tendinous insertion of the sartorius and gracilis muscles.

A species of spurious or adventitious bursæ is apt, in peculiar situations, to be generated or called into action by pressure and friction; for example, in the integuments over the ball of the great toe, over the patellar ligament, and in the club-foot, where it rests upon the ground. That on the tibial or inner side of the great toe seems gradually to multiply, or rather one bursal pouch is formed upon another, constituting what is named a bunion. The lateral ligament of the joint has a compact laminated structure, the laminæ dense towards the bone, and less so towards the skin, as if bursæ had successively formed, or were forming, as fast as the outer one was obliterated. In many instances, the head of the metatarsal bone is changed, the cartilaginous surface being covered with warty granulations. The tumour inflames, becomes exceedingly troublesome, and even causes lameness; it occasionally suppurates, involves the joint of the toe, and leads to amputation. At first, by fomentation and rest, and afterwards, the application of the nitrate of silver in the dry state, and the wearing of a wide shoe, with the sole thicker towards the tibial aspect of the foot, all these evils may be averted."

We may observe, that the affections of several of the bursæ are often mistaken, indeed more often than the contrary. The surgeon should be well aware where bursæ naturally exist, particularly such bursæ as are in the neighbourhood of joints. We have more than once seen disease of the sub-deltoidean bursa (not unfrequently a result of rheumatic fever) mistaken for disease of the shoulder. A provincial physician, of some eminence, pronounced a case of rheumatic disease of the sub-psoas bursa, morbus coxarius. We have twice, once during life, and once after death, seen disease of the complicated bursa beneath the obturatur internus, as it winds round the pully of the ischium. During life the case was thought sciatica, or disease of the hip-joint. We repeat, that surgeons are not sufficiently acquainted with the anatomy of the bursæ. The suprà patellar, and some of the other superficial bursæ are familiar to them: but the deeper ones, and especially the articular, are very imperfectly understood.

6. *Malformation of the Shoulder and Hip-joints.*—We extract some observations, which may be new to some of our readers, on these subjects.

"The shoulder-joint is sometimes so malformed, that the arm and hand are in a state of permanent pronation or supination, and were it not for other malformations, one would suppose the joint had been luxated at birth. I have not been able to get a *post-mortem* examination of this malformation. The treatment consists in applying pasteboard splints.

The whole arm is occasionally so shrivelled as to resemble the fin of a fish.

A superfluity of digits is no uncommon malformation, particularly two thumbs; in this case the outer pollex should be removed by amputation.

The hip-joint is sometimes deficient in its acetabulum, the head of the os femoris resting on the dorsum of the os ilium, nearer its crista than in the normal state. This is an imperfect formation of the os innominatum from some obstacle to the evolution of the bones, and no original luxation of the joint. The children born with this malformation have been in good health, but there is a marked disproportional breadth of the hips, with projection of the trochanters, and obliquity of the thigh-bones. As the child advances and begins to walk, the deformity increases; and still more so, when the pelvis begins to enlarge, and the child undergoes longer and more fatiguing exercise. Then the balancing of the trunk on the pelvis, its anterior inclination, a projecting of the belly, apparent shortening of stature, circular movements of the transverse diameter of the pelvis, want of fixity of the heads of the ossa femorum, alternate movements of elevation and descent of the head of the thigh-bone along the dorsum of the os ilium, all begin to be manifest.

When the sexual characters begin to develop themselves, the increase of the pelvis, more rapid and conspicuous in the girl than in the boy, renders in her the effects of this malformation much more apparent. Young females, from the play of the heads of the ossa femorum, become almost incapable of any great exertion, the heads ascending nearly to the cristæ of the ossa ilium; in those who are corpulent, pregnant, or dropsical, it amounts to an impossibility. The only palliative remedy is a broad belt round the pelvis, with iron supports down to the feet."

7. *Circular and Flap Amputations.*—The Edinburgh surgeons are known to be *Flappists.* Mr. Lizars makes no sort of hesitation in putting the circular mode hors de combat. "The circular incision," says he, "is now abandoned, and if applicable any where, it is to that of the arm between elbow and shoulder-joints; the first incision should sweep down to the bone, as done in time of Celsus, and revived by Dupuytren, and not layer after layer of skin, superficial and deep muscles, as recommended by Allanson and Hey."

This is more positive than convincing. The circular method is very far from abandoned. It is almost universally practised in London, and extensively in France, to say nothing of other parts of the world. Malgaigne, whose Manual of Operative Surgery is in every body's hands, and is the best work of the kind, admits that, on the whole, the circular method is the best. So far as our own experience goes, we prefer it, as a general mode of proceeding.

8. *The Tourniquet superseded.*—"The tourniquet is now only used by operators of the old school, or those of the modern ignorant of anatomy." p. 206.

This is de trop.

We shall notice the succeeding part of our dexterous friend's system of surgery, when it appears. Mr. Lizars has acquired a high reputation as an operator and a teacher, and his opinions on many points are necessarily worth knowing.

---

The work of Dr. Burns is of a more elaborate character than that of Mr. Lizars. It is not simply devoted to the obvious phenomena of disease and to the treatment required for them; but it treats at length of the principles both of pathology and treatment, those generalizations so important yet so difficult to draw.

Mr. Burns remarks, in his Preface, that a work on surgery to be comprehensive, must be divided into three parts.

First, a detail and exposition of those particular actions and conditions which are connected with injuries and operations in general, and of those doctrines and principles which furnish the rules of practice. Second, a minute and distinct description, of the relative situation, and particular connexion, of parts which may become the seat of disease, or the subject of operation. This constitutes what has been called Surgical Anatomy, and it is just as essential to the surgeon for the purpose of information and direction, as maps and charts are to the traveller and navigator. Third, an inquiry into the nature and treatment of individual diseases and injuries, and the best and safest way, of performing particular operations.

The first volume is devoted to the first of these objects. It comprises eleven chapters, which are headed, in succession, Preliminary Remarks on Action—Of Inflammation—Of Mortification—Of Suppuration—Of Ulceration—Of Tumors—Of Adhesion—Of Hæmorrhage and Wounded Arteries—Of Aneurysm and the Artificial Means of restraining Hæmorrhage—Of certain States connected with, or produced by, Wounds and Injuries—Of the Management of the Constitution under Disease and Injury.

The second volume carries out the remainder of our author's plan, and embraces the other objects proposed by him. The volume itself is intended, as he informs us, for two purposes :—First, to be a guide, or assistance, to the student, in the dissecting-room, enabling him, with one of the ordinary manuals, to trace the different parts of the body, with a view to their practical importance, and to acquiring a facility of finding them, when necessary, in the living subject. If employed for this purpose, it will be much for his advantage, that he mark the differences which may exist, in the individual he is examining. Second, to assist the surgeon in studying the nature of injuries, the relations of tumors, and in planning his operations.

It contains five chapters—the first, of the Anatomy of the Neck—the second, of the Anatomy of the Head—the third, of the Anatomy of the Clavicular Region, and Upper Extremity—the fourth, of the Pelvic Region—the fifth, of the Anatomy of the Inferior Extremity.

In a short notice it is impossible to present either a criticism or an analysis of so extensive a work. The first volume being mainly dedicated to principles, their comprehensive characters forbid a barren sketch of them. The second volume being filled with details, their extent interferes similarly with the reviewer. The only mode we can adopt for giving an idea of the nature of the volumes and the manner in which the subjects are handled, is to take a chapter in the first volume, and enumerate the main points that it discusses, and then to take a chapter in the second volume, and expose its entrails in the same manner.

We will take the tenth chapter in the first volume. Its heads are—of shock and its different kinds—exhaustion—rallying—reaction—acute sensation or pain—irritation—different states of danger, and the principle to be followed in deciding on operations—fever—arteritis—phlebitis—inflamed lymphatics—neuritis—neuralgia.

Suppose we pitch on the second chapter in the second volume—on the Anatomy of the Head. It is devoted to the—tongue—its size, shape, &c.—structure—covering and papillæ—diseases—submaxillary gland—extirpation of—sublingual—disease of—tonsil and its diseases—parotid gland and its diseases—extirpation of—ear—nostril—lachrymal duct—frontal, and other sinuses—antrum and its diseases—orbit—lachrymal gland—eyelid—sac—nerves in the orbit—muscles of the face—articulation of the jaw, and dislocation—tumors—lips—facial artery—transversalis faciei, &c.—temporal and internal maxillary—epistaxis—opening the temporal artery—veins of the face—nerves—wounds.

In conclusion, we recommend those students and surgeons who wish to learn the sentiments of Dr. Burns, or to consult a very comprehensive treatise on the principles and practice of surgery, to consult the work itself.

---

### RECENT WORKS ON ANATOMY.

I. Manual of Descriptive and Pathological Anatomy. By *J. F. Meckel*, Professor of Anatomy at Halle, &c. Translated from the German into French, with additions and notes, by *A. J. L. Jourdan*, Member of the Royal Academy of Medicine at Paris, &c. and *G. Breschet*, Adjunct Professor of Anatomy at the School of Medicine, &c. Translated from the French, with Notes, by *A. S. Doane*, A.M., M.D. and others. In two vols. 12mo. 1838.

II. PRACTICAL AND SURGICAL ANATOMY. By *W. J. Erasmus Wilson*, Lecturer on Practical and Surgical Anatomy and Physiology. Illustrated with fifty engravings on wood, by Bagg. Small 8vo. pp. 492.
III. A TEXT BOOK OF HUMAN ANATOMY, DESIGNED TO FACILITATE THE STUDY OF THAT SCIENCE. By *Robert Hunter*, M.D., Professor of Anatomy, Andersonian University, Glasgow. Small 8vo. pp. 220, 1838.
IV. THE SCIENCE OF THE CEREBRO-SPINAL PHENOMENA ATTEMPTED. By *John S. Waugh*, M.D. Annan. London, 1838.
V. OUTLINES OF HUMAN OSTEOLOGY. By *F. O. Warde*. Renshaw, London, 1838.
VI. THE SURGICAL ANATOMY OF THE PERINÆUM. By *Thomas Morton*, formerly one of the House-Surgeons of the University College Hospital. Large 8vo. pp. 80. 4 Plates.
VII. QUAIN'S PLATES. Fasciculi 62 and 63.

THE list of anatomical works is startling. We are certainly growing an anatomical people. Let us look at the works seriatim.

1. The merits and defects of Meckle's treatise are so well known that it would be idle to descant on them. But the English reader is now, for the first time, put in possession of the work. This was a desideratum which has been supplied. So far as we have glanced at it, the translation seems to be a good one.

2. Mr. Wilson is well known in London as a zealous practical anatomist and teacher. His manual contains many useful hints in the way both of teaching and of learning. The following brief quotation will give an idea of his plan and his plans.

"The *Aorta* arises from the left ventricle, at the middle of the root of the heart. It ascends at first to the right, then curves backwards and to the left, and descends on the right side of the vertebral column to the fourth lumbar vertebra. Hence it is divided into the ascending, arch, and descending aorta.

RELATIONS.—The *Ascending aorta* has in relation with it, in front, the trunk of the pulmonary artery, thoracic fascia, and pericardium; behind, the right pulmonary veins and artery; to the right side, the right auricle and superior cava; and to the left, the left auricle and trunk of the pulmonary artery.

Plan of the *relations* of the ascending Aorta.

*In Front*,
Pericardium.
Thoracic fascia.
Pulmonary artery.

| *Right Side.* | | *Left Side.* |
|---|---|---|
| Superior cava. | Ascending aorta. | Pulmonary artery. |
| Right auricle. | | Left auricle. |

*Behind.*
Right pulmonary artery.
Right pulmonary veins."

3. Mr. Hunter's text book appears to be compiled with diligence and care.

4. There is no knowing where the cerebro-spinal system will carry us. The reflex function had gone pretty far when it led, in the hands of Mr. Grainger, to walking being merely the effect of the ground upon the shoe-leather. But Dr. Waugh, of Annan, rather bothers us. He has created a new science, with new names, and whoever unwarily falls into the middle of it, will assuredly find himself in a strange land, and be accosted in an unknown tongue. We extract, at random, a passage.

"It may now be known, then, that the unspecial perceptive oli-versal filaments, and the unspecial protective versal filaments, conducting unspecial influence from the convolutions of the right cerebral hemisphere, accompany each other from the places of anastomosis at the bottoms of these convolutions, to within a very short distance of the median plane of the cerebro-spinal apparatus. In the instance of the minor system of the corpus callosum, the two orders of unspecial versal filaments in question accompany each other as far as the curvilinear line on the right side of the median line of that corpus. In the instance of the fornix another portion of those same two orders of unspecial versal filaments accompany each other as far as its right side. In the case of the anterior commissure, the same accompaniment takes place, till where the fasciculus of the anterior commissure leaves the mass of the right hemisphere. It may here be observed, that the most beautiful analogy prevails between the unspecial and special versal apparatuses of the primary cerebro-spinal ganglions, which are subservient to perceptive consciousness. As the unspecial perceptive oli-versal filaments accompany the unspecial protective versal filaments, for a certain distance from the unspecial grey tissue of the cerebral convolutions of the same side on which they originate; so the special perceptive oli-versal filaments accompany the special protective versal filament, for a certain distance, greater or less according to circumstances, from the special grey tissue in which they originate. This beautiful analogy assures me of the truth of the positions which it embraces. Its simple elegance will not be fully perceived till we come to treat of the special versal apparatuses of perceptive consciousness in their order."

We tremble for the fate of the "oli-versal" filaments. Men, we fear, will be prejudiced against them.

5. Mr. Ward's is, after all, the best account, so far as it goes, of the bones, that has yet been published.

As a sample, we may take at hazard the following simple account of the motions of the ribs, and their effects on the capacity of the thorax.

"If we examine in a skeleton the hoop formed by a pair of true ribs and costal cartilages, we shall find that it is inclined in two senses: 1st, the suture hoop is inclined downward and forward, from its spinal to its sternal attachment, so that its anterior is lower than its posterior portion; 2dly, each lateral segment of the hook is inclined downward and outward from the median plane of the body, so that the middle of the shaft of each rib is below the middle of a straight line connecting its extremities. To bring a rib thus inclined into a horizontal position, two things would be necessary: 1st, its anterior extremity must be raised to a level with its posterior extremity; and then its middle portion must be raised to a level with its ends. It is evident that the former of these actions would carry forward the anterior extremity of the rib and the sternum; thereby increasing the antero-posterior diameter of the chest; and that the latter would carry outward the middle of the rib, thereby increasing the transverse diameter of the chest; so that, performed simultaneously, they would dilate the cavity in both directions at once. Now each rib is provided with muscles by which its inclination with regard to the spine, and its inclination with regard to the median plane, are simultaneously diminished, all its parts being brought nearer to the horizontal plane in which its posterior extremity is situated. When we add that these muscles act during inspiration, and that their antagonists restore the ribs and sternum to their former position during expiration, the alternate dilatation and contraction of the walls of the thorax, are sufficiently explained; and we may proceed to consider how the same mobile walls are enabled to fulfil the condition required during all the more forcible actions of the muscles to which they give origin."

We have recommended this little work to our class.

6. Mr. Morton's book is also a good one. It comprises the recent information, and is likely to be very serviceable to young surgeons.

7. Of Quain's plates, we can only say that at present they maintain their character of excellence.

---

### NATURAL HISTORY.

I. A HISTORY OF BRITISH BIRDS. By *William Yarrell*, F.L.S., V.P.Z.S. Illustrated by a Wood-cut of each Species and numerous Vignettes. Part IX. Price 2s. 6d.

II. A GENERAL OUTLINE OF THE ANIMAL KINGDOM. By *Thomas Rymer Jones*, F.Z.S. Professor of Comparative Anatomy in King's College, London. Illustrated by numerous Engravings on Wood. Part II. October, 1838.

OF the first of these works it is almost needless to speak. It supports its own and its predecessor's character—no mean praise. We wish we could inoculate all our readers with a love of natural history. Let them get these works at all events. They are a pleasant addition to any library.

This part of Mr. Jones's work presents to us the polygastrica—the acalephæ and the sterilmintha. This work was much wanted, particularly by the younger members of the profession, whose attention we strongly direct to it. As a specimen of the style, we quote a short passage on the luminousness of the acalephæ.

" We are equally at a loss to account for the production of the irritating secretion in which the power of stinging seems to reside, but it is observed that the tentacula seem to be more specially imbued with it than other parts of the body. Perhaps the most remarkable property of the acalephæ is their phosphorescence, to which the luminosity of the ocean, an appearance especially beautiful in warm climates, is principally due. We have more than once witnessed this phenomenon in the Mediterranean, and the contemplation of it is well calculated to impress the mind with a consciousness of the profusion of living beings existing around us. The light is not constant, but only emitted when agitation of any kind disturbs the microscopic medusæ which crowd the surface of the ocean : a passing breeze, as it sweeps over the tranquil bosom of the sea, will call from the waves a flash of brilliancy which may be traced for miles; the wake of a ship is marked by a long track of splendour; the oars of your boat are raised dripping with living diamonds; and, if a little of the water be taken up in the palm of the hand and slightly agitated, luminous points are perceptibly diffused through it, which emanate from innumerable little acalephæ scarcely perceptible without the assistance of a microscope. All, however, are not equally minute; the Beroes, in which the cilia would seem to be most vividly phosphorescent, are of considerable size; the Cestum Veneris, as it glides rapidly along, has the appearance of an undulating riband of flame several feet in length; and many of the larger Pulmonigrade forms shine with such dazzling brightness, that they have been described by navigators as resembling 'white-hot shot' visible at some depth beneath the surface. This luminousness is undoubtedly dependent upon some phosphorescent secretion, but its nature and origin are quite unknown."

Mr. Jones has our best wishes for the success of his work. Mr. Van Voorst, the spirited publisher of the series, deserves every encouragement.

